# Ultrasound-guided minimally invasive autopsies: A protocol for the study of pulmonary and systemic involvement of COVID-19

**DOI:** 10.6061/clinics/2020/e1972

**Published:** 2020-05-18

**Authors:** Renata Aparecida de Almeida Monteiro, Amaro Nunes Duarte-Neto, Luiz Fernando Ferraz da Silva, Ellen Pierre de Oliveira, Jair Theodoro Filho, Glaucia Aparecida Bento dos Santos, Ilka Regina Souza de Oliveira, Thais Mauad, Paulo Hilário do Nascimento Saldiva, Marisa Dolhnikoff

**Affiliations:** I Brazilian Image Autopsy Study Group (BIAS), Departamento de Patologia, Faculdade de Medicina FMUSP, Universidade de Sao Paulo, Sao Paulo, SP, BR.; II Departamento de Cardiopneumologia, Instituto do Coracao (InCor), Faculdade de Medicina FMUSP, Universidade de Sao Paulo, Sao Paulo, SP, BR.; III Departamento de Radiologia e Oncologia, Faculdade de Medicina FMUSP, Universidade de Sao Paulo, Sao Paulo, SP, BR.

## INTRODUCTION

Over the last few decades, there has been a dramatic decrease in the number of hospital autopsies conducted around the world. However, autopsies are important for increasing our understanding of novel diseases. For example, autopsies were of vital importance in understanding acquired immunodeficiency syndrome (AIDS) and H1N1. There is a lack of literature on autopsies conducted on patients that have died of COVID-19 despite the high number of deaths worldwide. This is probably due to the risk of contagion, lack of skilled autopsy pathologists, and lack of adequately equipped services.

To prepare for the current pandemic and the potential high demand for advanced ventilation support of critically ill patients, our University Hospital, Hospital das Clínicas da Universidade de São Paulo, Brazil, dedicated all of its 900 beds (700 semi-intensive, 200 intensive) to treat patients with COVID-19 on March 24, 2020. Unfortunately, a substantial number of deaths are still occurring.

As part of the Department of Pathology, we have an autopsy service that has been in existence since 1924. Even though the number of autopsies performed at our institution has also decreased over time, 400 autopsies every year are still performed. In addition, the department provides training for residents in pathology from around Brazil. Several research projects are currently being conducted by the department, one of which, the Plataforma de Imagem na Sala de Autópsia (PISA) project, aims to examine the various imaging technologies that support the new concept of minimally invasive autopsies (MIA). 

Post mortem tissue sampling can help to guide the clinical management of patients with COVID-19, and understand the biology of the interaction between this novel infectious agent and the target cells and tissues. Ultrasound-based MIA (MIA/US) was used during the recent 2018 yellow fever epidemic in São Paulo and was found to have full diagnostic agreement with conventional autopsy (1). Our autopsy service decided to conduct MIA/US on patients that had died of COVID-19 due to “closed body autopsies” being considered significantly less risky in comparison to conventional autopsy. The first autopsy was performed on March 18, 2020. So far (April 29, 2020), we have evaluated 31 cases, all of which fulfilled the following requirements: ethical board approval, written consent from the next-of-kin, requested by the clinical team, and availability of our team.

## AUTOPSY PROCEDURE

We used a portable SonoSite M-Turbo R (Fujifilm, Bothell, WA, USA) ultrasound with C60x (5-2 MHz Convex) multi-frequency broadband transducers and DICOM^R^ standard images. This particular transducer was employed as lower frequency ultrasound waves permit a more in-depth visualization of all the organs, and can show pulmonary parenchyma. For superficial structures such as salivary glands and testis, we used the HFL38X (13-6 MHz Linear) transducer since the higher frequency results in a higher image resolution. Tissue puncture and collection was made using Tru-Cut^R^ semi-automatic 14G coaxial needles that were 20 cm long. Our protocol includes extensive sampling (multiple fragments of each organ, stored in formalin, glutaraldehyde, and deep freeze) of the lungs, heart, liver, kidneys, spleen, testis, skin, skeletal muscle (femoral quadriceps and intercostal muscle), bone marrow, salivary glands, brain, and intestines.

## CONCLUSION

MIA/US can be used to help clarify the pathogenesis of new infectious agents. The procedure was adopted in our hospital during the COVID-19 pandemic. Tissues from deceased patients were used to characterize the pathologic aspects of respiratory failure as well as the systemic organic manifestations of COVID-19, providing useful information for staff dealing with critically ill patients (2). Tissues collected from the different organs have been stored in a biorepository at our institution to be used for molecular studies. MIA/US is an alternative autopsy method that can be used in high contagion situations.
